# *Pneumo*Wiki: a pan-genome-based database for the pathogen *Streptococcus pneumoniae*

**DOI:** 10.1128/spectrum.02957-25

**Published:** 2026-03-20

**Authors:** Henry Mehlan, Stephanie Hirschmann, Larissa M. Busch, André Hennig, Kay Nieselt, Uwe Völker, Sven Hammerschmidt, Ulrike Mäder

**Affiliations:** 1 Department of Functional Genomics, Interfaculty Institute for Genetics and Functional Genomics, University Medicine Greifswald 60634, Greifswald, Germany; 2 Department of Molecular Genetics and Infection Biology, Interfaculty Institute for Genetics and Functional Genomics, University of Greifswald 26552, Greifswald, Germany; 3 Interfaculty Institute for Bioinformatics and Medical Informatics, University of Tübingen 9188, Tübingen, Germany; Emory University School of Medicine, Atlanta, Georgia, USA

**Keywords:** *Streptococcus pneumoniae*, database, pan-genome, genome annotation, *Pneumo*Wiki

## Abstract

The Gram-positive bacterium *Streptococcus pneumoniae* is a major human pathogen that exhibits more than 100 different capsular serotypes and considerable genomic variability. *S. pneumoniae* is also an important model organism for basic and biomedical research, with a number of strains with different characteristics being used. To address this, we have created the manually curated pan-genome database *Pneumo*Wiki (https://pneumowiki.med.uni-greifswald.de) that integrates genomic data of 43 *S. pneumoniae* strains with various aspects of functional annotation. All data relating to a specific gene and gene product are compiled on a *Gene pag*e. In addition to the gene and protein sequences and annotation from NCBI RefSeq, this includes, for example, data on gene expression, protein function, and localization. The information is supplemented with links to the data sources as well as to other databases and relevant literature. The basic concept of *Pneumo*Wiki is the interlinked presentation of pan-genome-based and strain-specific information. Therefore, register tabs with strain names at the top of the *Gene pages* allow switching between orthologous genes and the corresponding pan-genome page. The pan-genome gene pages contain a summary with functional information and the occurrence of the gene, an ortholog table for the 43 *S. pneumoniae* strains, a multiple-strain genome viewer as well as a protein sequence alignment. *Pneumo*Wiki was developed as a user-friendly tool and is available free of charge. The data collected in *Pneumo*Wiki are accessible *via* various download options. Overall, *Pneumo*Wiki is a resource for the research community that supports the analysis and interpretation of experimental data and, in particular, enables the integration of the knowledge available for different *S. pneumoniae* strains.

**IMPORTANCE:**

Databases that integrate and curate the large amounts of genomic data and functional information available for well-studied organisms play an increasingly important role in many areas of life sciences, including infection research. *Streptococcus pneumoniae* is a major human pathogen associated with high morbidity and mortality rates. The species exhibits high variability in its genome sequence, and a number of strains with different characteristics are used in pneumococcal research. We created the wiki-type database *Pneumo*Wiki to provide researchers with curated, up-to-date information on a range of pneumococcal genomes, focusing on the individual genes and encoded proteins and their functional annotation. The key feature of *Pneumo*Wiki is the pan-genome approach, which allows combining information of individual strains, thereby supporting the analysis of experimental data and comparisons between studies using different strains. In this way, the database becomes a valuable research tool that can contribute to a better understanding of pneumococcal physiology.

## INTRODUCTION

The amount and complexity of biological information is increasing rapidly. The underlying technological developments, particularly in the field of high-throughput methods, are considered to have great potential for infection research (1). However, in order to exploit this potential, the wealth of data and knowledge must be easily accessible, with databases on human pathogens playing an important role in this context (2–4).

The Gram-positive bacterium *Streptococcus pneumoniae* (the pneumococcus) is an opportunistic human pathogen that is responsible for high numbers of deaths worldwide, but it is also a common colonizer of the upper respiratory tract, mainly the nasopharynx. Triggered by certain host and environmental factors, the transition from asymptomatic colonization to infection can occur (5). *S. pneumoniae* is a leading cause of pneumonia, acute otitis media, and invasive diseases such as septicemia and meningitis worldwide. It is the most common bacterial pathogen causing community-acquired pneumonia, which is associated with a high mortality rate in developing countries, especially in infants and young children (6). One million children under the age of five die of pneumococcal disease every year, as estimated by the WHO. Furthermore, *S. pneumoniae* is the fourth most common pathogen in terms of deaths associated with antimicrobial resistance (7). One of the most important virulence factors of *S. pneumoniae* is the polysaccharide capsule that protects the bacteria from host immune defenses (8). While the capsule-based conjugate vaccines provide effective protection against pneumococcal infections, immunity is limited to serotypes included in the vaccine and leads to an increase in the prevalence of non-vaccine serotypes, mainly through serotype replacement (9, 10). Classification into capsule types is based on serology, and the structure of the capsular polysaccharide is determined by the *cps* locus. More than 100 different capsule types have been identified (10, 11; https://www.pneumogen.net/gps/#/serobank#serotypes).

*S. pneumoniae* is also one of the most important model organisms for studies on bacterial genetics and cell biology, pathogenesis, and host immune responses (12–14). These extensive studies have contributed to the characterization of the function of many genes, and genome-wide analyses have further improved the functional annotation of pneumococcal genes. These studies include, for example, the analysis of the transcriptome under infection-relevant and *in vivo* conditions (e.g., 15–17) as well as transposon insertion sequencing (Tn-seq) and CRISPR interference (CRISPRi) screens (18, 19). Important *S. pneumoniae* strains frequently used in studies of pneumococcal pathogenesis are TIGR4 (20) and D39. The strain D39 was originally isolated by Avery in 1916 (21). A detailed genome annotation of this strain performed by the Veening group was made available through the PneumoBrowse database in 2018 (22). It includes the annotation of new protein-coding genes and non-coding RNAs, transcriptional start sites, and operon structures. PneumoBrowse 2 provides not only updated genome annotation for strain D39 but also ChIP-seq data, ribosome profiling data, and CRISPRi-seq gene essentiality data (23). Genome sequences and annotations of 18 additional *S. pneumoniae* strains, including TIGR4, were also added to PneumoBrowse 2. The D39 strain whose genome was sequenced by the Veening group and serves as the basis for PneumoBrowse was designated D39V (22). The genome sequences of D39 (21) and D39V show some differences, which are described in detail by Slager et al. (22).

The pneumococcus has a relatively small genome with approximately 2.2 million base pairs and 2,200 protein-coding genes. The species shows significant genomic variability and plasticity, which is primarily achieved through natural competence and homologous recombination (24). In particular, mobile genetic elements (MGEs) contribute to this genomic variability and to the spread of antimicrobial resistance genes (14). For understanding the evolution and epidemiology of *S. pneumoniae*, the definition of pneumococcal lineages plays an important role. Based on thousands of genome sequences, strains have been classified into Global Pneumococcal Sequence Clusters (GPSCs) (25). Sequencing projects also provide information about the pan-genome of the species, which comprises the core genome with genes present in all strains, the accessory genome containing genes present in a subset of strains, and genes unique to single strains (26). The estimated pan-genome of *S. pneumoniae* comprises 5,000 to 7,000 clusters of orthologs (27). Currently, around 300 fully annotated genome sequences of *S. pneumoniae* strains are available in the NCBI RefSeq database.

Comprehensive functional annotation of bacterial genomes requires the integration of genomic data with bioinformatic predictions, existing knowledge, and experimental data sets. In particular, databases are essential that collect and curate the large amounts of genomic and functional information available for well-studied organisms (28). These resources, which integrate various kinds of data from different sources, support basic and applied research as well as functional annotation of the organism. They play such an important role because it is too time-consuming for individual laboratories to compile and maintain data collections containing gene-specific information required for the analysis of genome-scale data sets. The most comprehensive and most-used database for Gram-positive bacteria is *Subti*Wiki (29) dedicated to the functional annotation of the model organism *Bacillus subtilis*. It provides detailed information on the genes and proteins of strain 168, which is the most widely used laboratory strain of *B. subtilis. Subti*Wiki was created in 2008 (30) and since then continuously updated, with new data types added to the database (e.g., 31, 32). A related project was implemented with *Aureo*Wiki (33), a wiki-type database for the major Gram-positive pathogen *Staphylococcus aureus*, which developed into a comprehensive resource widely used by *S. aureus* researchers. Importantly, in contrast to *Subti*Wiki, it is based on a pan-genome approach to take account of the genetic variability that particularly plays a role in connection with pathogenic bacteria.

Here, we present *Pneumo*Wiki, a manually curated database focused on the functional annotation of *S. pneumoniae*, which contains genomic data and functional information obtained from relevant databases, bioinformatic predictions, and literature searches. As with *Aureo*Wiki, the special feature of *Pneumo*Wiki is its underlying pan-genome approach. The pan-genome was built from 43 pneumococcal genomes belonging to 13 serotypes using the latest versions of genome annotations from the NCBI RefSeq database. Orthologous genes of the individual strains are linked by a common identifier and a species-wide unified gene name and are displayed on the pan-genome pages. Information on all genes and gene products of the individual strains is compiled on the *Gene pages*, with register tabs allowing the user to switch directly between the orthologous genes of the individual strains. As experience with *Aureo*Wiki has shown, *Pneumo*Wiki will support the analysis and interpretation of experimental data as well as comparisons between studies using different strains. By combining gene and protein information of individual strains based on the *S. pneumoniae* pan-genome, the database also promotes the integration of available knowledge, thereby contributing to the functional annotation of pneumococcal genomes and to a better understanding of the physiology of this important human pathogen.

## RESULTS AND DISCUSSION

### Implementation of the pan-genome approach

**Fig 1:**
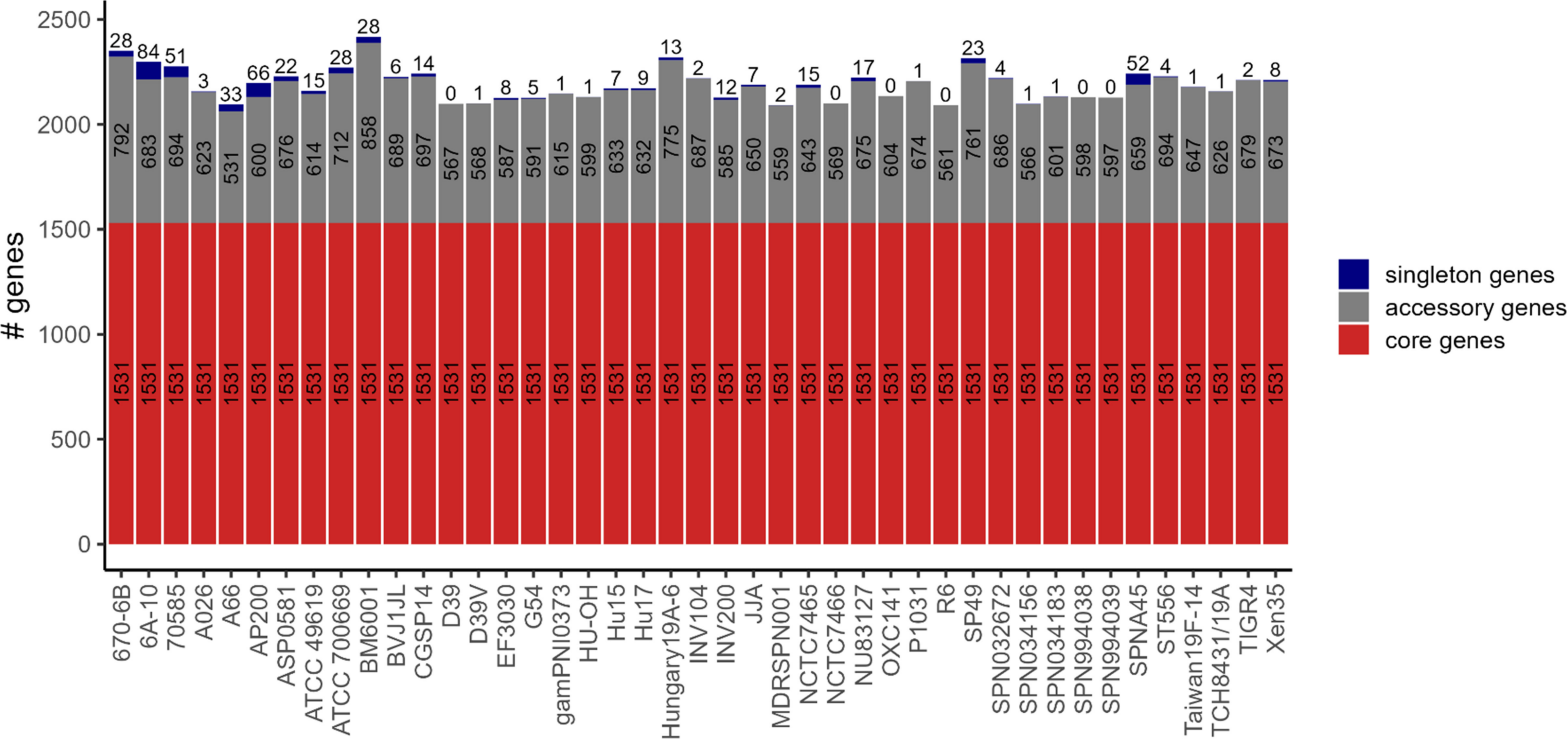
Numbers of core genes, accessory genes, and singleton genes in the 43 *S. pneumoniae* strains included in the pan-genome. Strains are sorted according to the *Pneumo*Wiki pan-genome page.

The *S. pneumoniae* pan-genome, which serves as the structural basis of *Pneumo*Wiki, was created using 43 fully annotated genomes from the NCBI RefSeq database as outlined in Materials and Methods. These 43 pneumococcal strains mainly comprise a set of 25 strains selected by Gamez et al. (34) to broadly represent different genetic backgrounds, as well as strains used in research on the biology and pathogenesis of pneumococci. This resulted in a pan-genome comprising 3,977 genes, of which 1,531 were defined as core genes. The individual strains contain between 531 and 858 accessory genes (Fig. 1), and 576 genes were unique to a single strain. The number of singleton genes in the individual genomes ranged from 0 to 84. It should be noted that the size of the pan-genome and the number of core genes naturally depends on the number of strains, the pan-genome analysis tool, and the DNA sequence-identity cutoffs used. However, a pan-genome of *S. pneumoniae* calculated on the basis of 208 strains covering 64 GPSCs and using three different tools yielded comparable numbers (35). Rosconi et al. determined an average pan-genome size of 4,640 genes and a core-genome of 1,411 genes.

The 43 *Pneumo*Wiki strains belong to 13 different serotypes and include well-characterized research strains such as TIGR4 (serotype 4), D39 (serotype 2), and its nonencapsulated derivative R6. Other strains include those that belong to the clinically relevant serotype 19F, such as the non-invasive strain EF3030 (36, 37) and BM6001, which is particularly rich in MGEs (38). The strain table is available in *Pneumo*Wiki and can be accessed from any page *via* the “Strains” link. In *Pneumo*Wiki, consistent terminology across the 43 *S. pneumoniae* strains is ensured by assigning a common identifier (pan ID or *pan locus tag*) and a species-wide uniform gene name (*pan gene symbol*) to each of the 3,977 pan-genes. The pan gene symbols are primarily based on the manually curated gene names of *S. pneumoniae* D39V (22, 23). For genes not present in strain D39V, gene symbols from the RefSeq annotation of other strains, starting with TIGR4, Hungary19A-6, and EF3030, serve as pan gene symbols. It is important to note that the gene names of D39V are the result of comprehensive automatic and manual curation, which also includes the scientific literature. Even in well-characterized model organisms, the function of many proteins is still poorly characterized or unknown. It is, therefore, important to take into account recent studies that have assigned functions to these proteins, which is also reflected in the assignment of gene symbols.

**Fig 2:**
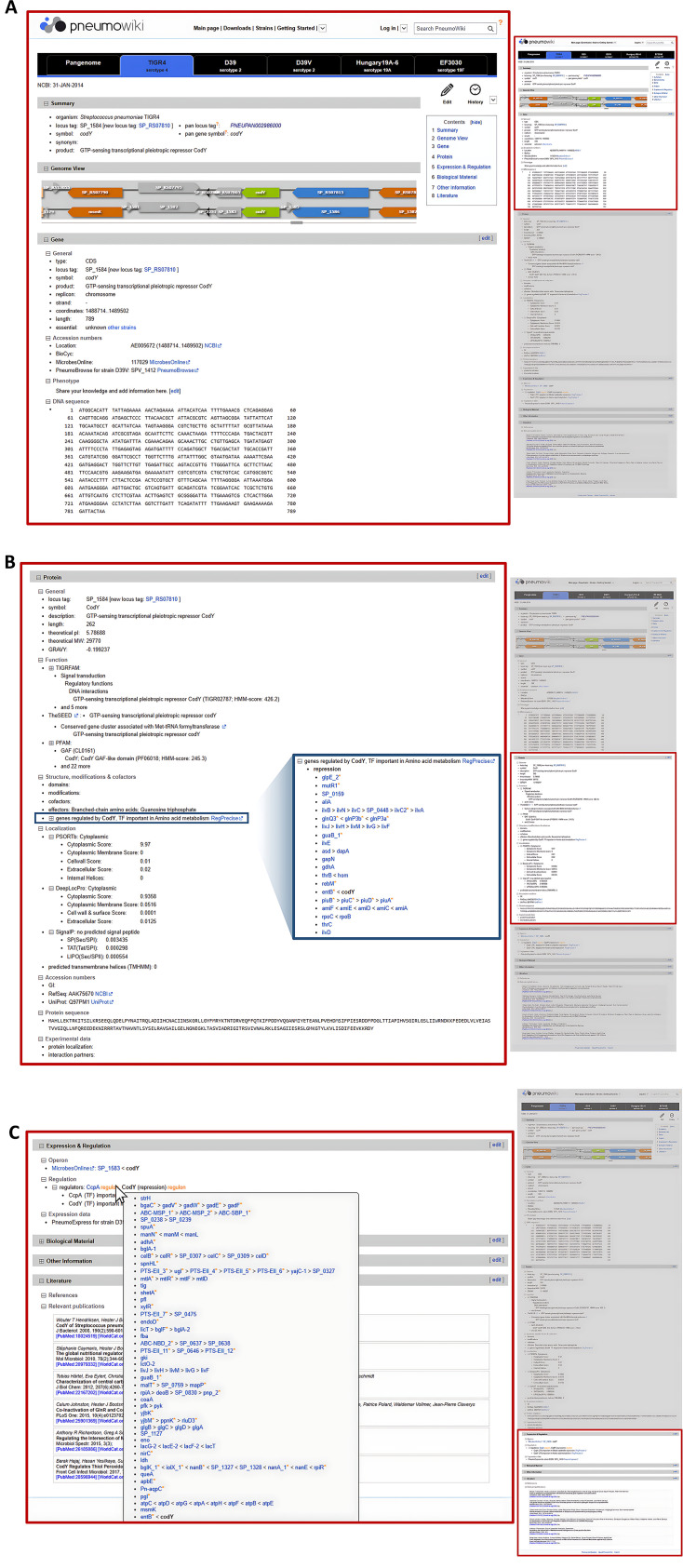
Example of a strain-specific *Gene page* of *Pneumo*Wiki. The *Gene page* contains information about the selected gene *codY* from *S. pneumoniae* TIGR4 and the corresponding protein CodY, which is a global regulator of stationary phase and virulence gene expression. All subsections can be expanded or collapsed by clicking on the plus/minus sign. (**A**) The register tabs at the top of the *Gene pages* allow switching to the pages of the orthologous genes of other *S. pneumoniae* strains and to the corresponding pan-genome page. The *Gene page* begins with the *Summary* section, which contains general gene information such as the locus tag, the gene name and the function of the gene product from the NCBI RefSeq annotation as well as the pan locus tag and the pan gene symbol. The *Genome View* displays both the NCBI GenBank and RefSeq annotations for the selected strain. This is followed by the *Gene* section. (**B**) The *Protein* section contains detailed information about the encoded protein including protein function assignments (based on TIGRFAMs, Pfam, and the SEED) and predicted subcellular localization (based on PSORTb, DeepLocPro, and SignalP). For regulatory proteins, the regulon and the link to RegPrecise or the literature source are displayed in the *Protein* section under the entry “genes regulated by …” (text with blue border; the expanded regulon list is shown as an insert). (**C**) The following sections of the *Gene page* contain data on gene expression and regulation, additional information, and references. As can be seen in the example of CcpA, moving the mouse pointer over “Regulon” displays a list of all genes that are also regulated by the respective transcription factor.

The pan-genome approach enables the interlinked presentation of orthologous genes to combine functional annotation data of different *S. pneumoniae* strains. In *Pneumo*Wiki, each pan-gene is represented by strain-specific *Gene pages* and a corresponding pan-genome page. Register tabs with the strain names at the top of the *Gene pages* (Fig. 2) allow the user to easily switch between pages of orthologous genes. The default setting shows five *S*. *pneumoniae* reference strains (TIGR4, D39, D39V, Hungary19A-6, and EF3030) that are frequently used in research. In addition, all data available for individual strains (e.g., on gene essentiality and regulation, see Materials and Methods) are directly accessible from the pages of all orthologous genes. In such cases, the user finds the entry “Data available for [name of the respective strain/s]”. By clicking on the strain name, the user is redirected to the *Gene page* with the corresponding data. On the pan-genome pages, the locus tags of the orthologous genes in all 43 strains are displayed and a complete ortholog table is available for download (as described below).

### The *Gene pages*

**Fig 3:**
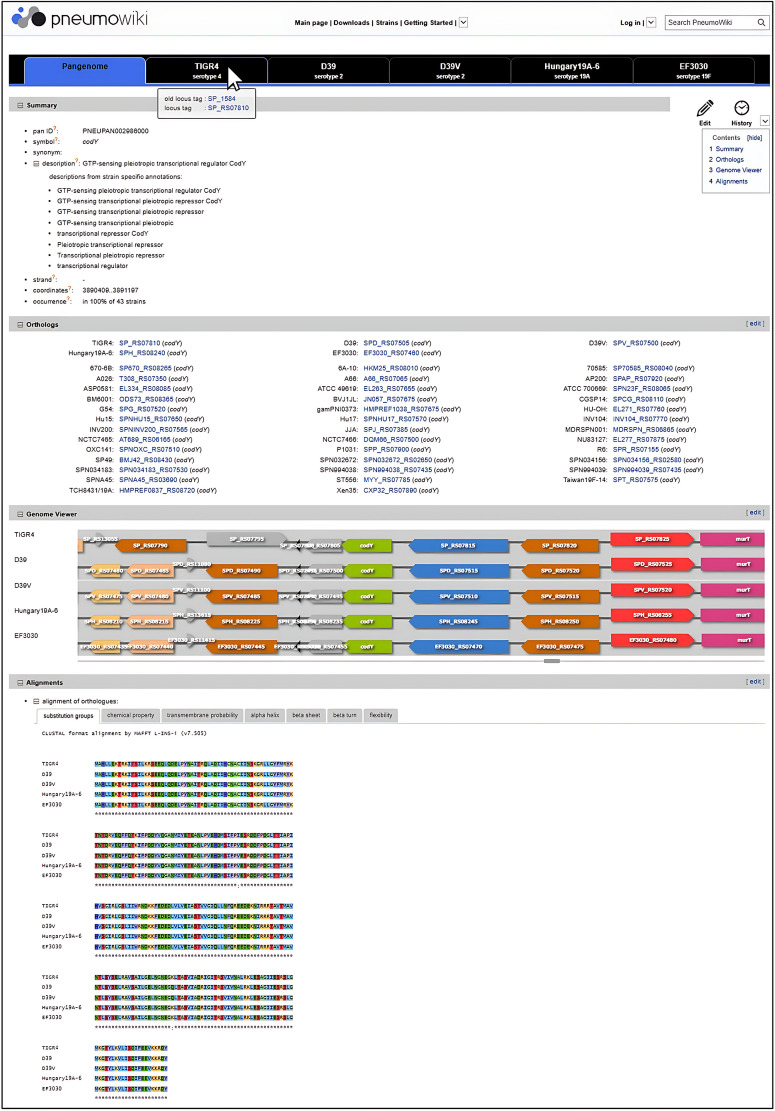
Example of a pan-genome page of *Pneumo*Wiki. The pan-genome pages contain combined information for the respective group of orthologous genes of 43 *S. pneumoniae* strains including the pan-genome identifier (pan ID), the species-wide unified gene name (symbol), the orthologs, the multiple-strain genome browser, and a protein sequence alignment. Unified gene names were assigned as pan gene symbol, which is provided in the *Summary* section of the pan-genome page as well as of the strain-specific *Gene pages*. In the *Orthologs* section, the 43 *S. pneumoniae* strains are listed in a fixed order and, if the gene is present in the respective strain, the locus tag and strain-specific gene name (if assigned) from the NCBI RefSeq annotation are shown.

*Gene pages* are provided for each gene present in 1 of the 43 *S. pneumoniae* genomes. In addition to the gene and protein sequences and annotation from RefSeq, the *Gene pages* contain a wealth of functional data on, for example, gene essentiality, protein function and localization, and transcriptional regulation. An example of a *Pneumo*Wiki *Gene page* is presented in Fig. 2. The *Summary* section at the top of each page contains the locus tag, the gene name and function of the gene product from the RefSeq annotation, as well as the pan locus tag and the pan gene symbol. This is followed by the *Genome View*, in which the genome browser is initially set to the position of the respective gene. The genome position can be changed by dragging the slider. The colors of the gene arrows represent the gene functional categories, as described in Materials and Methods. Of note, the genome browser combines the GenBank annotation (corresponding to the annotation submitted to NCBI) and the RefSeq annotation for the selected strain. For the RefSeq annotation, the new prokaryotic genome annotation pipeline introduced by the NCBI RefSeq project in 2015 (39) is used to annotate all prokaryotic genomes submitted to NCBI. This includes the re-annotation of all bacterial genomes, which were present in the RefSeq database before 2015, resulting in new “RS” containing gene identifiers (locus tags). The well-known gene identifiers of the GenBank annotations and earlier RefSeq annotations, which are widely used in publications and databases, are no longer supported by the NCBI resources. The direct comparison of the GenBank and RefSeq annotations in the *Genome View* facilitates understanding of differences in gene content and/or coordinates. In addition, *Pneumo*Wiki provides complete gene pages for both annotations. If the locus in the new annotation is a replacement of the original gene, the user can directly switch between the corresponding *Gene pages* by clicking on the second locus tag found in the *Summary* and *Gene* sections. The user can also choose between the two *Gene pages via* the register tabs specifying the strains (see Fig. 3).

The next section of the *Gene page* contains detailed information about the gene (*Gene* section, Fig. 2A). This section covers the basic information from the *Summary* section as well as the gene coordinates, gene length, essentiality, DNA sequence, and accession numbers used by external databases with corresponding links, including BioCyc (40) and PneumoBrowse 2 (23). The largest section, the *Protein* section (Fig. 2B), is dedicated to the encoded protein. Here, users can find the protein length, molecular weight, isoelectric point, catalyzed reaction, protein function assignments based on TIGRFAMs, Pfam, and the SEED (see Materials and Methods), subcellular localization, and other information. If the selected gene encodes a transcriptional regulator, the corresponding regulon members are displayed, as shown in Fig. 2B. As for the *Gene* section, the *Protein* section is concluded with database links (NCBI and UniProt) and the protein sequence. Functional annotations based on complementary classification algorithms (TIGRFAMs, Pfam, and the SEED) can support the elucidation of protein functions in the case of proteins with poorly characterized or unknown function. For the sake of conciseness, by default, the lists of assigned predicted functions according to TIGRFAMs and Pfam are collapsed and show only the hit with the best HMM-score but can be expanded by clicking on the plus sign.

The following section of the *Gene page* (*Expression and regulation*, Fig. 2C) provides information related to regulation and gene expression. This includes the predicted operon structure obtained from MicrobesOnline (41), regulation by transcription factors (Table 1), and a link to the respective gene expression data in PneumoExpress (15). For the TIGR4 strain, additional data on transcription start sites and operon structures determined experimentally by Warrier et al. (42) are provided.

**TABLE 1:** Transcription factor regulons collected in *Pneumo*Wiki^*a*^

Regulator (pan gene symbol)	Regulator (alternative name)	Type	Biological process	Target operons	Reference
AdcR		TF	Zinc homeostasis	6	RegPrecise (43)
AgaR		TF	N-acetylgalactosamine utilization	2	RegPrecise (43)
ArgR1	ArgR	TF	Arginine biosynthesis/degradation	7	RegPrecise (43)
ArgR2		TF	Arginine biosynthesis/degradation	7	RegPrecise (43)
AhrC		TF	Arginine biosynthesis/degradation	7	RegPrecise (43)
BirA		TF	Biotin biosynthesis	1	RegPrecise (43)
CcpA		TF	Global catabolite repression	73	RegPrecise (43)
CelR		TF	Cellobiose utilization	2	RegPrecise (43)
CmbR		TF	Cysteine metabolism	7	RegPrecise (43)
CodY		TF	Amino acid metabolism	20	RegPrecise (43)
ComE		Response regulator	Competence	9	(44)
ComX1	ComX	RNAP sigma factor	Competence	14	(44)
CopY	CopR	TF	Copper homeostasis	1	RegPrecise (43)
CtsR		TF	Heat shock response	5	RegPrecise (43)
FabT		TF	Fatty acid biosynthesis	3	RegPrecise (43)
FruR		TF	Fructose utilization	1	RegPrecise (43)
FucR		TF	Fucose utilization	2	RegPrecise (43)
GalR		TF	Galactose utilization	2	RegPrecise (43)
GlnR		TF	Nitrogen assimilation	5	RegPrecise (43)
GntR	SPy1285	TF	Membrane transport	3	RegPrecise (43)
HrcA		TF	Heat shock response	2	RegPrecise (43)
LacR		TF	Lactose/galactose utilization	2	RegPrecise (43)
MalR		TF	Maltose/maltodextrin utilization	6	RegPrecise (43)
MntR		TF	Manganese homeostasis	2	RegPrecise (43)
MtaR	CmhR	TF	Methionine metabolism	7	RegPrecise (43)
MtlR		TF	Mannitol utilization	1	RegPrecise (43)
NagR		TF	N-acetylglucosamine utilization	3	RegPrecise (43)
NanR		TF	Sialic acid utilization	3	RegPrecise (43)
NiaR		TF	NAD biosynthesis	3	RegPrecise (43)
NmlR		TF	Carbonyl stress response	1	RegPrecise (43)
NrdR		TF	Deoxyribonucleotide biosynthesis	2	RegPrecise (43)
PflR		TF	Formate metabolism	3	RegPrecise (43)
PipR		TF	Phage infection protein	2	RegPrecise (43)
PtvR	YwzG	TF	Membrane transport	1	RegPrecise (43)
PurR		TF	Purine metabolism	4	RegPrecise (43)
RegR		TF	Hyaluronate utilization	3	RegPrecise (43)
Rex		TF	Energy metabolism	9	RegPrecise (43)
ScrR		TF	Sucrose utilization	2	RegPrecise (43)
SczA		TF	Zinc resistance	2	RegPrecise (43)
SusR	BfrR	TF	Fructooligosaccharides utilization	1	RegPrecise (43)
TreR		TF	Trehalose utilization	2	RegPrecise (43)
UlaR	SgaR	TF	Ascorbate utilization	1	RegPrecise (43)
XylR	CelQ	TF	Cellobiose utilization	2	RegPrecise (43)

^*a*^ TF, transcription factor; RNAP, RNA polymerase.

Data on transcription factor regulons are retrieved from the RegPrecise database (43) and from published literature (Table 1). Currently, most regulons originate from RegPrecise, while the ComX and ComDE regulons are based on the work of Slager et al. (44). If data on gene expression regulation are available for the respective gene, the transcription factor(s) and their general functions are displayed. When the user moves the mouse pointer over “Regulon”, a list of all genes regulated by these transcription factors is also displayed, as shown in Fig. 2C for the CcpA regulon.

All data on the *Gene pages* are provided with links to the external data sources, including databases and published literature. The references are indicated by a book symbol, and details of the corresponding publication are displayed by mouse-over. The *Literature* section at the bottom of the *Gene page* (Fig. 2C) contains these *References* and a list of additional *Relevant publications*.

### The pan-genome pages

Each *S. pneumoniae* pan-gene is represented by strain-specific gene pages and a corresponding pan-genome page containing comparative information. The pan-genome page (Fig. 3) begins with a *Summary* section containing the pan locus tag, the pan gene symbol, (putative) functions of the encoded protein extracted from the RefSeq annotations of the 43 strains, the multiple genome alignment coordinates (see Materials and Methods), and the gene occurrence frequency expressed as percentage of 43 strains. In the following *Orthologs* section, the 43 *S. pneumoniae* strains are listed in a fixed order and, if the gene is present in the respective strain, the locus tag is shown together with the strain-specific gene name, if assigned, from the RefSeq annotation. The next two sections, *Genome Viewer* and *Alignments*, refer to the five *S*. *pneumoniae* reference strains displayed in the default setting. The multiple-strain genome viewer has the same features and uses the same color scheme based on functional categories as the strain-specific *Genome View* displayed on the *Gene pages*. Finally, a protein sequence alignment generated by the MAFFT program (45) is provided. This can be displayed using different color schemes, for example, by coloring the amino acids according to their chemical properties.

### Data accessibility and updates

**Fig 4:**
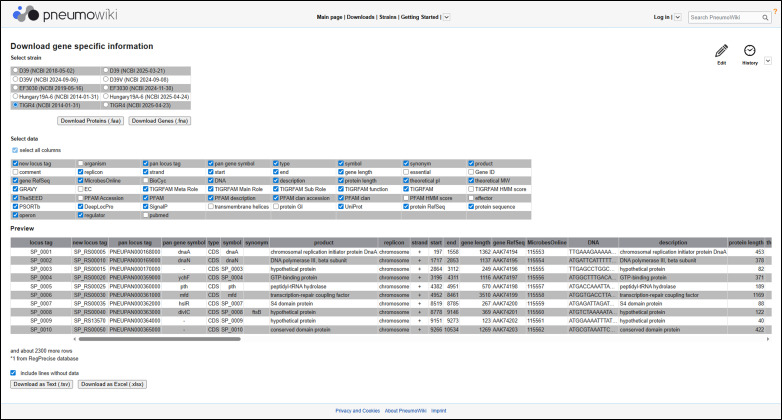
Example of a download page for gene-specific information in *Pneumo*Wiki. After selecting one reference strain and annotation, the desired data can be selected from a menu. By selecting “all columns,” the resulting table will contain more than 50 entries per gene, representing the majority of the data from the *Gene pages*. The customized table can be downloaded as .tsv or .xlsx file.

Data accessibility is supported by various download options to meet the requirements of bioinformatic analyses. The *Downloads* page can be accessed from any *Pneumo*Wiki page via the corresponding link at the top of the window. By selecting “gene-specific information” on the *Downloads* page, users are redirected to a page (Fig. 4) where they can select (i) the strain (TIGR4, D39, D39V, Hungary19A-6, or EF3030) and annotation (GenBank or RefSeq) and (ii) the data to be downloaded. By selecting “all columns,” the resulting table will contain more than 50 entries per gene, representing most of the data from the *Gene pages*. This also includes functional classification and regulon assignment, which are essential for the analysis of genome-scale data sets. In addition, FASTA files for gene and protein sequences are available for download for the five reference strains. The second link on the *Downloads* page allows users to download the “orthologue table”. This table contains the common identifiers (pan locus tags) of the 3,977 *S. pneumoniae* pan-genes in the first column, followed by separate columns for each of the 43 (or selected) strains listing the corresponding strain-specific locus tags.

The content of *Pneumo*Wiki will be constantly updated in order to keep pace with updates of the NCBI RefSeq annotations and other databases that are queried for data on *S. pneumoniae* genes and proteins. Regular updates concern, for example, relevant publications and links to external databases. In the case of significant changes to data stored in *Pneumo*Wiki, for example, with regard to the RefSeq annotations, the necessary updates are performed using an automated pipeline. In addition, the maintainers of *Pneumo*Wiki will update the functional annotation of gene products, especially those with so far unknown function, on the basis of current publications, with new pan gene symbols being assigned together with the corresponding literature references. The same applies to new findings on the assignment of genes to transcription factor regulons. In this way, *Pneumo*Wiki contributes to the continuous improvement of functional annotation of *S. pneumoniae* based on published data.

Information preceded by a filled bullet point on the *Gene pages* originates from the database behind *Pneumo*Wiki and cannot be edited by the user. In particular, all sequence-based information needs to be consistently maintained in accordance with the NCBI RefSeq annotations. The same holds true for links to other databases. However, users can contribute additional data and information *via* the [Edit] link. In particular, placeholders were inserted that request input from the user to supplement the existing information, for example, regarding the phenotypes of gene knockout or overexpression mutants. Information added by users to the *Gene pages* appears without bullet points or with open bullet points.

### Conclusions and perspectives

In line with the genomic variability and plasticity of the species *S. pneumoniae*, a number of strains with different characteristics are used for research on pneumococcal pathogenesis and in molecular and cell biology. The wiki-based database *Pneumo*Wiki was created to provide the research community with curated, up-to-date information on a range of pneumococcal genomes, with a focus on the individual genetic elements and their functional annotation. The decisive factor is the pan-genome approach, which enables the integration of data and information available for different strains and supports the analysis of experimental data, especially from omics experiments.

Future efforts will focus, in particular, on adding further data on transcriptional regulation and including non-coding RNAs. The availability of comprehensive information on gene regulation is an important aspect of functional annotation. In the current version of *Pneumo*Wiki, 43 transcriptional regulators and their target genes, mainly from RegPrecise, are recorded (Table 1). However, regulons controlled by response regulators of two-component systems (TCSs) (46) are not covered by RegPrecise and are therefore severely underrepresented even though several studies have been conducted in *S. pneumoniae* (e.g., 47, 48). For this reason, we are currently reviewing relevant publications to extract additional data on transcriptional regulation, with a particular focus on TCSs, and to make this information available for *Pneumo*Wiki. In the next step, we will comprehensively extract regulon information from published data sets to supplement the data available in RegPrecise, both in terms of missing target genes of transcription factors and unrecorded regulators. Several studies have also identified small regulatory RNAs (sRNAs) in *S. pneumoniae*, especially in the context of virulence and competence control (e.g., 49–51). As already implemented in *Aureo*Wiki, we will create special pages for non-coding RNAs, divided into housekeeping RNAs and sRNAs. These pages will contain, in particular, a description of the general function and a list of target genes with the literature references and links to the corresponding *Gene pages* as well as other relevant publications on the respective non-coding RNA.

Finally, experimental data for *S. pneumoniae*, especially omics data, will be added to *Pneumo*Wiki for targeted queries and interactive visualizations. This will also include graphical representations of condition-specific gene expression on the respective *Pneumo*Wiki *Gene pages*. The planned data integration initially covers transcriptome and proteome data, in particular, for the serotype 4 strain TIGR4 and the serotype 19F strain EF3030 under various growth and infection-relevant conditions.

## MATERIALS AND METHODS

### Creation of the *S. pneumoniae* pan-genome

The *S. pneumoniae* pan-genome was created on the basis of a SuperGenome, analogous to its sibling platform *Aureo*Wiki (33, 52, 53). For this purpose, 43 sequenced and annotated *S. pneumoniae* genomes from the NCBI RefSeq database were processed. The complete list of strains included can be found at https://pneumowiki.med.uni-greifswald.de/Strains. First, a global multiple sequence alignment was generated using progressiveMauve (54). Based on this alignment, a SuperGenome was computed representing the multiple alignment to which a common coordinate system is added (53). This involves calculating a mapping between the coordinates of each individual genome to a position in this common coordinate system. On the basis of overlapping genes in the coordinate system of the SuperGenome, homologous genes were assigned to ortholog gene groups. Pair-wise homologous genes were considered with at least 50% identity on DNA level between both genes and an overlap ratio of at least 0.4, where the overlap between two genes is defined as the number of base pairs with the same SuperGenome position mappings and the overlap ratio refers to the total length of the shorter gene. In a second step, ortholog gene groups located between core genes, which do not overlap in the SuperGenome due to non-optimal alignment, are merged based on sequence similarity on amino acid level. Ortholog gene groups are referred to as pan-genome genes.

### The *Pneumo*Wiki platform

Like the *Aureo*Wiki, *Pneumo*Wiki is based on a MediaWiki platform, which has been supplemented with extensions for gene- and protein-specific data, for example, for displaying the *Genome View*. All collected data are stored in a separate database and embedded in the wiki’s pages using user-defined tags. This architecture allows modified and new data to be made available in the *Pneumo*Wiki without having to edit each page individually. In addition, all data are available for download. The visual and plain text editors provided by MediaWiki allow users to add further data and information.

### Data

The data presented in *Pneumo*Wiki are based on the sequences and gene annotations of NCBI RefSeq (39, 55). Data on operon prediction (MicrobesOnline [41]), gene essentiality (19, 56), and gene regulation extend the data basis. Transcription factor regulons were obtained from the RegPrecise database (43) and extracted from the literature. For *S. pneumoniae* TIGR4, RegPrecise provides manually curated reconstructions of transcription factor regulons that include the regulated genes and downstream operon genes. For 10 additional strains included in *Pneumo*Wiki, “propagated” regulons were obtained from RegPrecise that list the genes directly preceded by a putative transcription factor binding site. For these strains, we transferred the operons (transcription units) from the corresponding TIGR4 regulons based on orthologous genes in the *S. pneumoniae* pan-genome.

Functional assignments are generated as follows: The EC numbers of the proteins extracted from NCBI RefSeq were extended with the corresponding enzyme names and reaction equations available at ExPASy (http://enzyme.expasy.org/). The SEED database (57) provides a protein function prediction based on curated subsystems (sets of related functional roles) across many genomes (https://www.theseed.org/wiki/Home_of_the_SEED). If available, the subsystem name, subcategory, and category are displayed in a tree-like structure alongside the functional role. TIGRFAM is a database of manually curated protein family definitions (58) represented by trusted sequences, each protein family described by a Hidden Markov Model (HMM). These TIGRFAM HMMs are used to predict the function of proteins by comparing their sequences using the HMMR3 software package (59). The TIGRFAMs models for each protein are ordered by their HMM comparison score and displayed by the tree-like structure (main role, sub role, function) given for each TIGRFAM model, which has been extended by a meta role summarizing the related main roles. These seven meta roles are also used to color the genes in the genome viewer. Like the TIGRFAMs, the protein sequences were assigned to the Pfam protein families (60) and displayed according to the HMM score. In addition to displaying the Pfam annotation, any associated Pfam clan (families with a common evolutionary origin) is also displayed.

PSORTb (http://psort.org/psortb/) and DeepLocPro v1.0 (61; https://services.healthtech.dtu.dk/services/DeepLocPro-1.0/) were used to predict the subcellular localization of the proteins. If the prediction by PSORTb is not explicit, “unknown (no significant prediction)” is displayed. TMHMM (https://services.healthtech.dtu.dk/services/TMHMM-2.0/) was used to predict the number of transmembrane helices.

## Data Availability

The *Pneumo*Wiki database is available at https://pneumowiki.med.uni-greifswald.de.

## References

[1] Eckhardt M, Hultquist JF, Kaake RM, Hüttenhain R, Krogan NJ. 2020. A systems approach to infectious disease. Nat Rev Genet 21:339–354. doi:10.1038/s41576-020-0212-5 32060427 PMC7839161

[2] Elfmann C, Zhu B, Stülke J, Halbedel S. 2023. ListiWiki: a database for the foodborne pathogen Listeria monocytogenes. Int J Med Microbiol 313:151591. doi:10.1016/j.ijmm.2023.151591 38043216

[3] Skrzypek MS, Binkley J, Binkley G, Miyasato SR, Simison M, Sherlock G. 2017. The Candida Genome Database (CGD): incorporation of Assembly 22, systematic identifiers and visualization of high throughput sequencing data. Nucleic Acids Res 45:D592–D596. doi:10.1093/nar/gkw924 27738138 PMC5210628

[4] Winsor GL, Griffiths EJ, Lo R, Dhillon BK, Shay JA, Brinkman FSL. 2016. Enhanced annotations and features for comparing thousands of Pseudomonas genomes in the Pseudomonas genome database. Nucleic Acids Res 44:D646–D653. doi:10.1093/nar/gkv1227 26578582 PMC4702867

[5] Weiser JN, Ferreira DM, Paton JC. 2018. Streptococcus pneumoniae: transmission, colonization and invasion. Nat Rev Microbiol 16:355–367. doi:10.1038/s41579-018-0001-8 29599457 PMC5949087

[6] Wahl B, O’Brien KL, Greenbaum A, Majumder A, Liu L, Chu Y, Lukšić I, Nair H, McAllister DA, Campbell H, Rudan I, Black R, Knoll MD. 2018. Burden of Streptococcus pneumoniae and Haemophilus influenzae type b disease in children in the era of conjugate vaccines: global, regional, and national estimates for 2000–15. Lancet Glob Health 6:e744–e757. doi:10.1016/S2214-109X(18)30247-X 29903376 PMC6005122

[7] Naghavi M, Vollset SE, Ikuta KS, Swetschinski LR, Gray AP, Wool EE, Robles Aguilar G, Mestrovic T, Smith G, Han C, et al.. 2024. Global burden of bacterial antimicrobial resistance 1990–2021: a systematic analysis with forecasts to 2050. Lancet 404:1199–1226. doi:10.1016/S0140-6736(24)01867-1 39299261 PMC11718157

[8] Hyams C, Camberlein E, Cohen JM, Bax K, Brown JS. 2010. The Streptococcus pneumoniae capsule inhibits complement activity and neutrophil phagocytosis by multiple mechanisms. Infect Immun 78:704–715. doi:10.1128/IAI.00881-09 19948837 PMC2812187

[9] Feikin DR, Kagucia EW, Loo JD, Link-Gelles R, Puhan MA, Cherian T, Levine OS, Whitney CG, O’Brien KL, Moore MR, Serotype Replacement Study Group. 2013. Serotype-specific changes in invasive pneumococcal disease after pneumococcal conjugate vaccine introduction: a pooled analysis of multiple surveillance sites. PLoS Med 10:e1001517. doi:10.1371/journal.pmed.1001517 24086113 PMC3782411

[10] Ganaie FA, Beall BW, Yu J, van der Linden M, McGee L, Satzke C, Manna S, Lo SW, Bentley SD, Ravenscroft N, Nahm MH. 2025. Update on the evolving landscape of pneumococcal capsule types: new discoveries and way forward. Clin Microbiol Rev 38:e0017524. doi:10.1128/cmr.00175-24 39878373 PMC11905375

[11] Ganaie F, Saad JS, McGee L, van Tonder AJ, Bentley SD, Lo SW, Gladstone RA, Turner P, Keenan JD, Breiman RF, Nahm MH. 2020. A new pneumococcal capsule type, 10D, is the 100th serotype and has a large cps fragment from an oral Streptococcus. mBio 11:e00937-20. doi:10.1128/mBio.00937-20 32430472 PMC7240158

[12] Henriques-Normark B, Tuomanen EI. 2013. The pneumococcus: epidemiology, microbiology, and pathogenesis. Cold Spring Harb Perspect Med 3:a010215. doi:10.1101/cshperspect.a010215 23818515 PMC3685878

[13] Hiller NL, Orihuela CJ. 2024. Biological puzzles solved by using Streptococcus pneumoniae: a historical review of the pneumococcal studies that have impacted medicine and shaped molecular bacteriology. J Bacteriol 206:e0005924. doi:10.1128/jb.00059-24 38809015 PMC11332154

[14] Santoro F, Iannelli F, Pozzi G. 2019. Genomics and genetics of Streptococcus pneumoniae. Microbiol Spectr 7. doi:10.1128/microbiolspec.gpp3-0025-2018 PMC11315030 31111814

[15] Aprianto R, Slager J, Holsappel S, Veening JW. 2018. High-resolution analysis of the pneumococcal transcriptome under a wide range of infection-relevant conditions. Nucleic Acids Res 46:9990–10006. doi:10.1093/nar/gky750 30165663 PMC6212715

[16] D’Mello A, Riegler AN, Martínez E, Beno SM, Ricketts TD, Foxman EF, Orihuela CJ, Tettelin H. 2020. An in vivo atlas of host-pathogen transcriptomes during Streptococcus pneumoniae colonization and disease. Proc Natl Acad Sci USA 117:33507–33518. doi:10.1073/pnas.2010428117 33318198 PMC7777036

[17] Kimaro Mlacha SZ, Romero-Steiner S, Hotopp JCD, Kumar N, Ishmael N, Riley DR, Farooq U, Creasy TH, Tallon LJ, Liu X, Goldsmith CS, Sampson J, Carlone GM, Hollingshead SK, Scott JAG, Tettelin H. 2013. Phenotypic, genomic, and transcriptional characterization of Streptococcus pneumoniae interacting with human pharyngeal cells. BMC Genomics 14:383. doi:10.1186/1471-2164-14-383 23758733 PMC3708772

[18] Jana B, Liu X, Dénéréaz J, Park H, Leshchiner D, Liu B, Gallay C, Zhu J, Veening J-W, van Opijnen T. 2024. CRISPRi–TnSeq maps genome-wide interactions between essential and non-essential genes in bacteria. Nat Microbiol 9:2395–2409. doi:10.1038/s41564-024-01759-x 39030344 PMC11371651

[19] van Opijnen T, Camilli A. 2012. A fine scale phenotype-genotype virulence map of a bacterial pathogen. Genome Res 22:2541–2551. doi:10.1101/gr.137430.112 22826510 PMC3514683

[20] Tettelin H, Nelson KE, Paulsen IT, Eisen JA, Read TD, Peterson S, Heidelberg J, DeBoy RT, Haft DH, Dodson RJ, et al.. 2001. Complete genome sequence of a virulent isolate of Streptococcus pneumoniae. Science 293:498–506. doi:10.1126/science.1061217 11463916

[21] Lanie JA, Ng WL, Kazmierczak KM, Andrzejewski TM, Davidsen TM, Wayne KJ, Tettelin H, Glass JI, Winkler ME. 2007. Genome sequence of Avery’s virulent serotype 2 strain D39 of Streptococcus pneumoniae and comparison with that of unencapsulated laboratory strain R6. J Bacteriol 189:38–51. doi:10.1128/JB.01148-06 17041037 PMC1797212

[22] Slager J, Aprianto R, Veening JW. 2018. Deep genome annotation of the opportunistic human pathogen Streptococcus pneumoniae D39. Nucleic Acids Res 46:9971–9989. doi:10.1093/nar/gky725 30107613 PMC6212727

[23] Janssen AB, Gibson PS, Bravo AM, de Bakker V, Slager J, Veening J-W. 2025. PneumoBrowse 2: an integrated visual platform for curated genome annotation and multiomics data analysis of Streptococcus pneumoniae. Nucleic Acids Res 53:D839–D851. doi:10.1093/nar/gkae923 39436044 PMC11701578

[24] Salvadori G, Junges R, Morrison DA, Petersen FC. 2019. Competence in Streptococcus pneumoniae and close commensal relatives: mechanisms and implications. Front Cell Infect Microbiol 9:94. doi:10.3389/fcimb.2019.00094 31001492 PMC6456647

[25] Gladstone RA, Lo SW, Lees JA, Croucher NJ, van Tonder AJ, Corander J, Page AJ, Marttinen P, Bentley LJ, Ochoa TJ, et al.. 2019. International genomic definition of pneumococcal lineages, to contextualise disease, antibiotic resistance and vaccine impact. EBioMedicine 43:338–346. doi:10.1016/j.ebiom.2019.04.021 31003929 PMC6557916

[26] Medini D, Donati C, Tettelin H, Masignani V, Rappuoli R. 2005. The microbial pan-genome. Curr Opin Genet Dev 15:589–594. doi:10.1016/j.gde.2005.09.006 16185861

[27] Hiller NL, Sá-Leão R. 2018. Puzzling over the pneumococcal pangenome. Front Microbiol 9:2580. doi:10.3389/fmicb.2018.02580 30425695 PMC6218428

[28] Oliver SG, Lock A, Harris MA, Nurse P, Wood V. 2016. Model organism databases: essential resources that need the support of both funders and users. BMC Biol 14:49. doi:10.1186/s12915-016-0276-z 27334346 PMC4918006

[29] Elfmann C, Dumann V, van den Berg T, Stülke J. 2025. A new framework for SubtiWiki, the database for the model organism Bacillus subtilis. Nucleic Acids Res 53:D864–D870. doi:10.1093/nar/gkae957 39441067 PMC11701700

[30] Lammers CR, Flórez LA, Schmeisky AG, Roppel SF, Mäder U, Hamoen L, Stülke J. 2010. Connecting parts with processes: SubtiWiki and SubtiPathways integrate gene and pathway annotation for Bacillus subtilis. Microbiology (Reading) 156:849–859. doi:10.1099/mic.0.035790-0 19959575

[31] Mäder U, Schmeisky AG, Flórez LA, Stülke J. 2012. SubtiWiki—a comprehensive community resource for the model organism Bacillus subtilis. Nucleic Acids Res 40:D1278–D1287. doi:10.1093/nar/gkr923 22096228 PMC3245094

[32] Zhu B, Stülke J. 2018. SubtiWiki in 2018: from genes and proteins to functional network annotation of the model organism Bacillus subtilis. Nucleic Acids Res 46:D743–D748. doi:10.1093/nar/gkx908 29788229 PMC5753275

[33] Fuchs S, Mehlan H, Bernhardt J, Hennig A, Michalik S, Surmann K, Pané-Farré J, Giese A, Weiss S, Backert L, Herbig A, Nieselt K, Hecker M, Völker U, Mäder U. 2018. AureoWiki - The repository of the Staphylococcus aureus research and annotation community. Int J Med Microbiol 308:558–568. doi:10.1016/j.ijmm.2017.11.011 29198880

[34] Gámez G, Castro A, Gómez-Mejia A, Gallego M, Bedoya A, Camargo M, Hammerschmidt S. 2018. The variome of pneumococcal virulence factors and regulators. BMC Genomics 19:10. doi:10.1186/s12864-017-4376-0 29298677 PMC5753484

[35] Rosconi F, Rudmann E, Li J, Surujon D, Anthony J, Frank M, Jones DS, Rock C, Rosch JW, Johnston CD, van Opijnen T. 2022. A bacterial pan-genome makes gene essentiality strain-dependent and evolvable. Nat Microbiol 7:1580–1592. doi:10.1038/s41564-022-01208-7 36097170 PMC9519441

[36] De S, Busch LM, Burchhardt G, Gesell Salazar M, Schlüter R, Steil L, Völker U, Hammerschmidt S. 2025. The global proteome of Streptococcus pneumoniae EF3030 under nutrient-defined in vitro conditions. Front Cell Infect Microbiol 15:1606161. doi:10.3389/fcimb.2025.1606161 40718681 PMC12289677

[37] Junges R, Maienschein-Cline M, Morrison DA, Petersen FC. 2019. Complete genome sequence of Streptococcus pneumoniae serotype 19F strain EF3030. Microbiol Resour Announc 8:e00198-19. doi:10.1128/MRA.00198-19 31072896 PMC6509521

[38] Colombini L, Cuppone AM, Tirziu M, Lazzeri E, Pozzi G, Santoro F, Iannelli F. 2023. The mobilome-enriched genome of the competence-deficient Streptococcus pneumoniae BM6001, the original host of integrative conjugative element Tn5253, is phylogenetically distinct from historical pneumococcal genomes. Microorganisms 11:1646. doi:10.3390/microorganisms11071646 37512819 PMC10383233

[39] Tatusova T, Ciufo S, Federhen S, Fedorov B, McVeigh R, O’Neill K, Tolstoy I, Zaslavsky L. 2015. Update on RefSeq microbial genomes resources. Nucleic Acids Res 43:D599–D605. doi:10.1093/nar/gku1062 25510495 PMC4383903

[40] Karp PD, Billington R, Caspi R, Fulcher CA, Latendresse M, Kothari A, Keseler IM, Krummenacker M, Midford PE, Ong Q, Ong WK, Paley SM, Subhraveti P. 2019. The BioCyc collection of microbial genomes and metabolic pathways. Brief Bioinform 20:1085–1093. doi:10.1093/bib/bbx085 29447345 PMC6781571

[41] Dehal PS, Joachimiak MP, Price MN, Bates JT, Baumohl JK, Chivian D, Friedland GD, Huang KH, Keller K, Novichkov PS, Dubchak IL, Alm EJ, Arkin AP. 2010. MicrobesOnline: an integrated portal for comparative and functional genomics. Nucleic Acids Res 38:D396–D400. doi:10.1093/nar/gkp919 19906701 PMC2808868

[42] Warrier I, Ram-Mohan N, Zhu Z, Hazery A, Echlin H, Rosch J, Meyer MM, van Opijnen T. 2018. The transcriptional landscape of Streptococcus pneumoniae TIGR4 reveals a complex operon architecture and abundant riboregulation critical for growth and virulence. PLoS Pathog 14:e1007461. doi:10.1371/journal.ppat.1007461 30517198 PMC6296669

[43] Novichkov PS, Kazakov AE, Ravcheev DA, Leyn SA, Kovaleva GY, Sutormin RA, Kazanov MD, Riehl W, Arkin AP, Dubchak I, Rodionov DA. 2013. RegPrecise 3.0 – A resource for genome-scale exploration of transcriptional regulation in bacteria. BMC Genomics 14:745. doi:10.1186/1471-2164-14-745 24175918 PMC3840689

[44] Slager J, Aprianto R, Veening JW. 2019. Refining the pneumococcal competence regulon by RNA sequencing. J Bacteriol 201:e00780-18. doi:10.1128/JB.00780-18 30885934 PMC6560143

[45] Katoh K, Standley DM. 2013. MAFFT multiple sequence alignment software version 7: improvements in performance and usability. Mol Biol Evol 30:772–780. doi:10.1093/molbev/mst010 23329690 PMC3603318

[46] Gómez-Mejia A, Gámez G, Hammerschmidt S. 2018. Streptococcus pneumoniae two-component regulatory systems: the interplay of the pneumococcus with its environment. Int J Med Microbiol 308:722–737. doi:10.1016/j.ijmm.2017.11.012 29221986

[47] McCluskey J, Hinds J, Husain S, Witney A, Mitchell TJ. 2004. A two-component system that controls the expression of pneumococcal surface antigen A (PsaA) and regulates virulence and resistance to oxidative stress in Streptococcus pneumoniae. Mol Microbiol 51:1661–1675. doi:10.1111/j.1365-2958.2003.03917.x 15009893

[48] Mohedano ML, Amblar M, de la Fuente A, Wells JM, López P. 2016. The response regulator YycF inhibits expression of the fatty acid biosynthesis repressor FabT in Streptococcus pneumoniae. Front Microbiol 7:1326. doi:10.3389/fmicb.2016.01326 27610104 PMC4996995

[49] Halfmann A, Kovács M, Hakenbeck R, Brückner R. 2007. Identification of the genes directly controlled by the response regulator CiaR in Streptococcus pneumoniae: five out of 15 promoters drive expression of small non-coding RNAs. Mol Microbiol 66:110–126. doi:10.1111/j.1365-2958.2007.05900.x 17725562

[50] Mann B, van Opijnen T, Wang J, Obert C, Wang Y-D, Carter R, McGoldrick DJ, Ridout G, Camilli A, Tuomanen EI, Rosch JW. 2012. Control of virulence by small RNAs in Streptococcus pneumoniae. PLoS Pathog 8:e1002788. doi:10.1371/journal.ppat.1002788 22807675 PMC3395615

[51] Tsui H-C, Mukherjee D, Ray VA, Sham L-T, Feig AL, Winkler ME. 2010. Identification and characterization of noncoding small RNAs in Streptococcus pneumoniae serotype 2 strain D39. J Bacteriol 192:264–279. doi:10.1128/JB.01204-09 19854910 PMC2798261

[52] Hennig A, Bernhardt J, Nieselt K. 2015. Pan-Tetris: an interactive visualisation for Pan-genomes. BMC Bioinformatics 16:S3. doi:10.1186/1471-2105-16-S11-S3 PMC4547177 26328606

[53] Herbig A, Jäger G, Battke F, Nieselt K. 2012. GenomeRing: alignment visualization based on SuperGenome coordinates. Bioinformatics 28:i7–i15. doi:10.1093/bioinformatics/bts217 22689781 PMC3371849

[54] Darling AE, Mau B, Perna NT. 2010. progressiveMauve: multiple genome alignment with gene gain, loss and rearrangement. PLoS One 5:e11147. doi:10.1371/journal.pone.0011147 20593022 PMC2892488

[55] Li W, O’Neill KR, Haft DH, DiCuccio M, Chetvernin V, Badretdin A, Coulouris G, Chitsaz F, Derbyshire MK, Durkin AS, Gonzales NR, Gwadz M, Lanczycki CJ, Song JS, Thanki N, Wang J, Yamashita RA, Yang M, Zheng C, Marchler-Bauer A, Thibaud-Nissen F. 2021. RefSeq: expanding the Prokaryotic Genome Annotation Pipeline reach with protein family model curation. Nucleic Acids Res 49:D1020–D1028. doi:10.1093/nar/gkaa1105 33270901 PMC7779008

[56] Thanassi JA, Hartman-Neumann SL, Dougherty TJ, Dougherty BA, Pucci MJ. 2002. Identification of 113 conserved essential genes using a high-throughput gene disruption system in Streptococcus pneumoniae. Nucleic Acids Res 30:3152–3162. doi:10.1093/nar/gkf418 12136097 PMC135739

[57] Overbeek R, Begley T, Butler RM, Choudhuri JV, Chuang H-Y, Cohoon M, de Crécy-Lagard V, Diaz N, Disz T, Edwards R, et al.. 2005. The subsystems approach to genome annotation and its use in the project to annotate 1000 genomes. Nucleic Acids Res 33:5691–5702. doi:10.1093/nar/gki866 16214803 PMC1251668

[58] Haft DH, Selengut JD, Richter RA, Harkins D, Basu MK, Beck E. 2013. TIGRFAMs and genome properties in 2013. Nucleic Acids Res 41:D387–D395. doi:10.1093/nar/gks1234 23197656 PMC3531188

[59] Finn RD, Clements J, Eddy SR. 2011. HMMER web server: interactive sequence similarity searching. Nucleic Acids Res 39:W29–W37. doi:10.1093/nar/gkr367 21593126 PMC3125773

[60] Finn RD, Coggill P, Eberhardt RY, Eddy SR, Mistry J, Mitchell AL, Potter SC, Punta M, Qureshi M, Sangrador-Vegas A, Salazar GA, Tate J, Bateman A. 2016. The Pfam protein families database: towards a more sustainable future. Nucleic Acids Res 44:D279–D285. doi:10.1093/nar/gkv1344 26673716 PMC4702930

[61] Moreno J, Nielsen H, Winther O, Teufel F. 2024. Predicting the subcellular location of prokaryotic proteins with DeepLocPro. Bioinformatics 40:btae677. doi:10.1093/bioinformatics/btae677 39540738 PMC11645106

